# Polycystic ovary syndrome (PCOS) affects relative embryo morphokinetics observed by time-lapse imaging: an observational study

**DOI:** 10.1007/s00404-026-08335-0

**Published:** 2026-02-08

**Authors:** Vera Monika Garçon, Jens Erik Dietrich, Thomas Strowitzki, Alexander Freis

**Affiliations:** 1 Department of Gynecological Endocrinology and Fertility Disorders, Heidelberg University Women’s Hospital, Heidelberg University, Im Neuenheimer Feld 440, 69120 Heidelberg, Germany; 2 Medicus Bergen, Bredalsmarken 15, 5006 Bergen, Norway

**Keywords:** Embryo development, Female infertility, Assisted reproduction, Intracytoplasmic sperm injection, In vitro fertilisation

## Abstract

**Purpose:**

To characterise the effect of polycystic ovary syndrome (PCOS) on embryo morphokinetics via time-lapse imaging, including absolute time points, relative time intervals, and ratios representing cleavage synchronicity.

**Methods:**

This single-centre retrospective observational study examined patients aged 18–45 years undergoing in vitro fertilisation/intracytoplasmic sperm injection with time-lapse imaging (09/2016–12/2019; *n* = 1433 two-pronuclear oocytes). A group with PCOS (*n* = 48 embryos) was compared to a control group with uterine, tubal factor or idiopathic infertility (*n* = 400 embryos). Times from the two-cell stage to blastocyst expansion, eight intervals for embryonic cell cycle (ECC) duration and synchronicity and four cleavage synchronicity (CS) and DNA replication time ratios were analysed.

**Results:**

PCOS patients were younger (*P* = 0.023) with higher anti-Müllerian hormone levels (*P* < 0.001) than controls. No statistically noticeable influence of PCOS on absolute times was observed. The intervals from the 3- to 4-cell (synchronicity of cell cycle 2, s2; *P* = 0.013), the 5- to 8-cell (synchronicity of cell cycle 3, s3; *P* = 0.032) and the 4- to 8-cell stage (ECC3; *P* = 0.043) were longer in the PCOS group. The relative CS ratio from the 2- to 8-cell stage (CS2-8) was lower (*P* = 0.003) and from the 2- to 4-cell stage (CS2-4) was higher (*P* = 0.001) in PCOS embryos.

**Conclusion:**

Whilst absolute times remained unaffected, relative morphokinetic intervals and ratios, potentially indicating poorer cleavage synchronicity, were altered in PCOS embryos. This is the first study examining the influence of PCOS on relative morphokinetic ratios.

What does this study add to the clinical work:

Relative morphokinetic embryo development intervals and ratios, representing cleavage synchronicity, were altered in PCOS embryos, whereas routinely assessed absolute morphokinetic time points remained unaffected. These findings suggest that relative morphokinetics, especially ratios, could contribute to identifying impaired cleavage patterns in clinical practice.

## Introduction

Time-lapse imaging (TLI) technology allows for continuous observation of preimplantation embryo development without disturbing culture conditions and has become an integral part in the practice of embryo assessment [1–3]. Rather than only analysing absolute morphokinetic time points, researchers attempted to enhance embryo assessment by introducing relative morphokinetic variables representing cleavage synchronicity [4, 5]. Meseguer et al. found the time intervals from the three-cell to four-cell stage (synchronicity of cell cycle 2, s2) and the two-cell to three-cell stage as predictive parameters of embryo implantation [6]. Dal Canto et al. observed significant differences in the intervals from the five- to eight-cell stage (synchronicity of cell cycle 3, s3) and the four- to eight-cell stage for embryos developing to blastocyst stage compared to embryos arresting after the eight-cell stage [7]. Cetinkaya et al. published four relative morphokinetic time ratios to investigate the synchronicity of cell cycles: the cleavage synchronicity (CS) ratio from the 2- to 8-cell (CS2-8), 4- to 8-cell (CS4-8) and 2- to 4-cell stage (CS2-4) as well as the DNA replication time ratio (DR). The ratios CS2-8, s3 and CS4-8 outperformed all absolute morphokinetic time points, yielding the highest area under the curve (AUC) for the prediction of blastocyst quality [8]. Despite their superiority in predicting blastocyst quality, there has been little further investigation into these ratios.

Embryo morphokinetics may be influenced not only by external factors, including in vitro culture conditions, but also by patients’ comorbidities [9]. Polycystic ovary syndrome (PCOS), affecting 4–21% of women worldwide, is a common indication for assisted reproductive technologies, as one of the leading causes of female infertility [10]. Previous studies comparing the morphokinetics of PCOS and non-PCOS embryos showed inconsistent results, ranging from no detectable differences in timings to either delayed or even accelerated absolute morphokinetic time points [11–14]. Whilst one study found that only relative ratios, rather than absolute timings, detected endometriosis-associated morphokinetic alterations, no research has yet focussed on the influence of PCOS on relative morphokinetic ratios [9]. Therefore, this paper aims to examine how not only absolute morphokinetic time points, but also time intervals and especially relative ratios, are altered in embryos of PCOS patients when compared to embryos of patients with uterine factor, tubal factor, or idiopathic infertility.

Some of the results of this study have previously been reported in the form of an abstract [15].

## Methods

### Study design and population

**Fig. 1:**
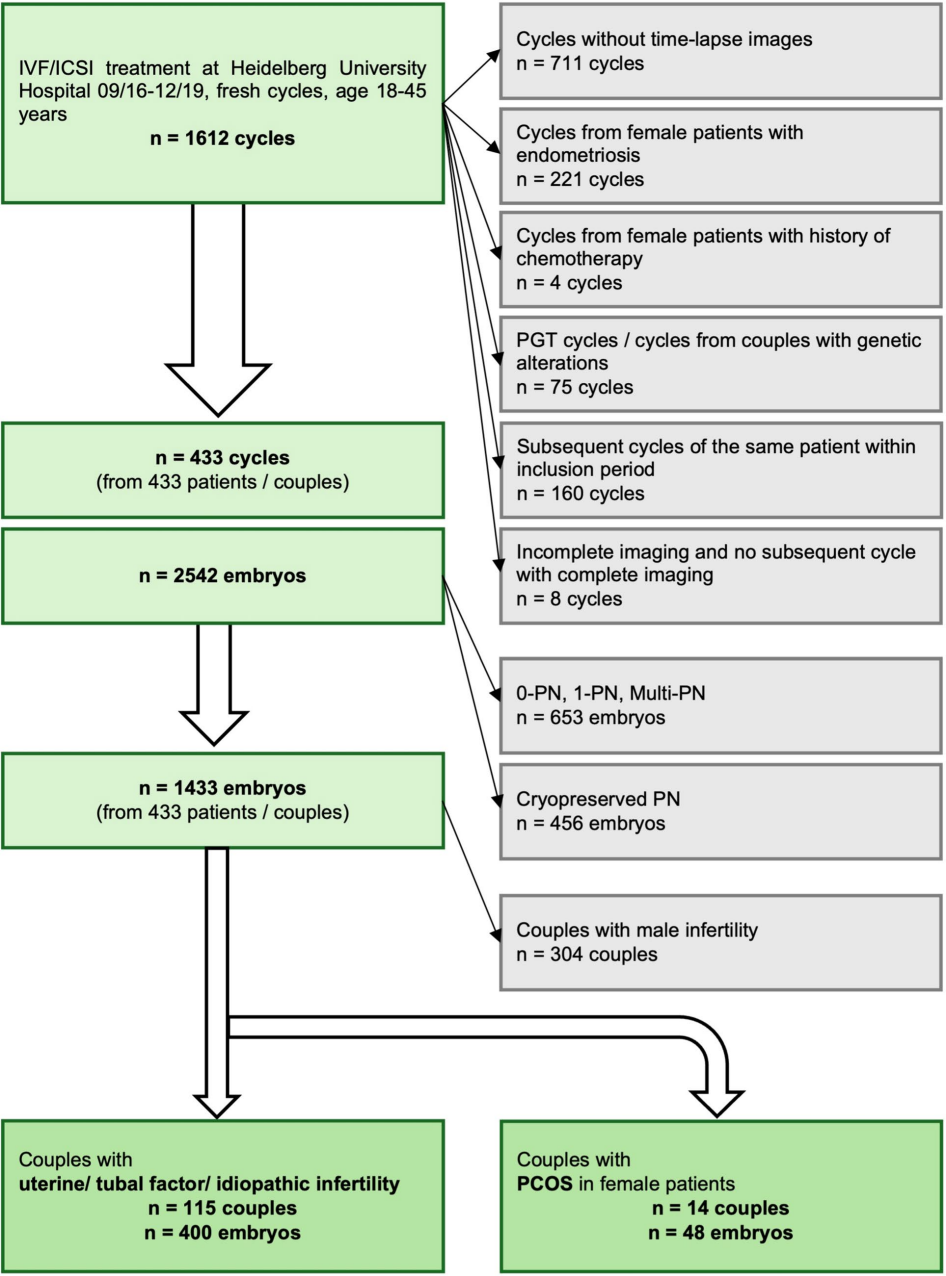
Flowchart for inclusion/exclusion of treatment cycles, couples and embryos. IVF, in vitro fertilisation; ICSI, intracytoplasmic sperm injection; PGT, preimplantation genetic testing; PN, (*n*-) pronuclear stage, PCOS, polycystic ovary syndrome

This study was a single-centre observational study conducted at the Department of Gynecological Endocrinology and Fertility Disorders of Heidelberg University Women’s Hospital, Germany. The study retrospectively analysed clinical and TLI data routinely collected between September 2016 and December 2019 [15]. Study data were drawn from *n* = 1612 in vitro fertilisation (IVF) and intracytoplasmic sperm injection (ICSI) fresh cycles (Fig. 1). The female study collective was 18–45 years old. All cycles without TLI were excluded. Cycles from female patients with endometriosis or with a history of chemotherapy were excluded from the study. All cycles with preimplantation genetic testing and all cycles of couples with genetic alterations were excluded from the study. Only one cycle of each couple with complete annotation of time-lapse images was included in the study. All two-pronuclear oocytes from these remaining cycles made a total of *n* = 1433 included fertilised oocytes (embryos) from *n* = 433 couples [15].

All couples in treatment for male factor infertility were excluded from the study. The remaining couples were split into a group in which female patients fulfilled the diagnostic criteria for PCOS (*n* = 14 couples, *n* = 48 embryos) versus a group that did not fulfil the diagnostic criteria and was in treatment for uterine factor, tubal factor, or idiopathic infertility (*n* = 115 couples, *n* = 400 embryos) [15]. Diagnostic criteria for PCOS were defined according to the 2003 Rotterdam Consensus workshop by the European Society of Human Reproduction and Embryology (ESHRE) and the American Society for Reproductive Medicine (ASRM) [16]. PCOS was diagnosed when other aetiologies were excluded and at least two of the following criteria were met:

1. Oligo- and/or anovulation
2. Clinical and/or biochemical hyperandrogenemia
3. Polycystic ovaries

### Ovarian stimulation

Controlled ovarian stimulation was performed according to the gonadotropin-releasing hormone-(GnRH)-antagonist and long GnRH-agonist protocol, depending on patients’ individual characteristics (e.g. PCOS, ovarian reserve). Both protocols were accepted, as a previous study from our clinic has not shown any difference in morphokinetics between agonist and antagonist regimens [17]. Oocyte retrieval was achieved under transvaginal ultrasound monitoring 34–36 h after ovulation induction with recombinant human chorionic gonadotropin (hCG).

### IVF/ICSI and embryo culture

IVF as well as ICSI and IVF/ICSI splitting cycles with non-androgenic indication were included in the study. Oocytes and embryos were cultured in CSCM-C (Irvine Scientific, 90,165) covered with paraffin oil (10100060A, Origo) at 37 °C in an atmosphere of 5.0% O_2_ within a pH range of 7.25–7.35. Oocytes were placed into individual wells of an EmbryoSlide culture dish (FT-S-ES-D, Vitrolife, Sweden), either directly after ICSI or after 16–18 h of insemination in case of IVF.

### Time-lapse imaging

Culture was performed in two EmbryoScope™ TLI incubators (ES-D2, Unisense FertiliTech A/S, now Vitrolife, Sweden). Images were taken every 10 min in seven focal planes and annotated by trained embryologists until the day of embryo transfer. All annotations were again retrospectively checked by only one person using EmbryoViewer® software (Version 7.7, Vitrolife) to minimise inter-observer variability. Morphokinetic time points and intervals were annotated according to Ciray et al. [4]. Morphokinetic time points from the fading of pronuclei (tPNf), through all cleavage stages beginning at the two-cell stage (t2, t3, t4, t5, t6, t7, t8, t9 +), to the initiation of compaction (tSC), morula stage (tM), blastulation (tSB), and expansion of the blastocyst (tEB) were annotated. The duration of the embryonic cell cycle (ECC) was calculated for the second (ECC2 = t4–t2) and third cycle (ECC3 = t8–t4). Furthermore, single cell cycles (cc) of the blastomeres were assessed for the second (cc2a = t3–t2; cc2b = t4–t2) and third (cc3a = t5–t4; cc3b = t6–t4; cc3c = t7–t4; cc3d = t8–t4) ECC. The synchronicity of cell cycle 2 (s2 = t4–t3) and synchronicity of cell cycle 3 (s3 = t8–t5) were assessed [4]. The four relative morphokinetic time ratios were calculated according to Cetinkaya et al. [8]:

- CS2-8 = ((t3–t2) + (t5–t4))/(t8–t2)
  o Ideal ratio tending to 1, worst ratio tending to 0;
- CS4-8 = (t8–t5)/(t8–t4)
  o Ideal ratio tending to 0, worst ratio tending to 1;
- CS2-4 = (t4–t3)/(t4–t2)
  o Ideal ratio tending to 0, worst ratio tending to 1;
- DR = (t3–t2)/(t5–t3).

### Statistical analysis

Statistics were performed using SPSS Statistics® (Version 27 for Windows 10, IBM). To account for different timings between IVF and ICSI cycles, absolute morphokinetic time points were normalised to tPNf. Patient and treatment characteristics were compared using the Mann–Whitney *U* test for continuous variables and Fisher’s exact test for categorical data [15]. The primary analysis for normalised time points, intervals and ratios was performed using a Generalised Linear Mixed Model (GLMM). This approach was chosen to account for the inherent hierarchical structure of the data (clustering of multiple embryos within a single patient), by treating the patient ID as a random effect [15]. Given the exploratory nature of this study and the lack of prior data for power calculations focussing on relative morphokinetics, the sample size was determined by the total number of available cases during the study period. To ensure the robustness of our findings, sensitivity analyses were conducted: a Mann–Whitney *U* test was performed to address potential violations of the Gaussian assumption in the GLMM due to small group sizes, and a *t* test was used to additionally assess the consistency of the observed effects. A *P* value < 0.05 was considered statistically noticeable. In accordance with the exploratory design of this study, *P* values are reported as a measure of evidence to generate hypotheses for future research rather than as formal confirmatory proof. No adjustment for multiple testing was performed and results should be interpreted as explorative.

## Results

### Patient- and treatment-related characteristics

PCOS patients were younger compared to non-PCOS controls (33.3 ± 4.6 years vs 36.2 ± 4.3 years, *P* = 0.023, Table 1) [15]. Serum levels of anti-Müllerian hormone (AMH) were higher in the PCOS group compared to the control group (7.5 ± 4.9 ng/ml vs 2.7 ± 2.0 ng/ml, *P* < 0.001, Table 1) [15]. Other patient-related characteristics did not differ noticeably between the groups (Table 1).

**Table 1:** Patient- and treatment-related characteristics

Characteristics		PCOS group		Control group		*P* value^a^
Patient related						
Female age [years]	*n*	14		115		
	Mean ± SD	33.3	± 4.6	36.2	± 4.3	**0.023**
Male age [years]	*n*	14		115		
	Mean ± SD	34.9	± 5.7	38.2	± 6.1	0.053
Female BMI [kg/m^2^]	*n*	14		115		
	Mean ± SD	25.0	± 5.4	23.8	± 4.5	0.567
AMH level [ng/ml]	*n*	13		108		
	Mean ± SD	7.5	± 4.9	2.7	± 2.0	** < 0.001**
Unsuccessfully transferred embryos^b^	*n*	14		115		
	Mean ± SD	2.1	± 3.6	2.1	± 3.6	0.936
Smoking	Non-smoker (%)	14	(100.0)	102	(88.7)	0.358
	Smoker (%)	0	(0.0)	13	(11.3)	
Hypothyroidism	No (%)	9	(64.3)	73	(63.5)	1.000
	Yes (%)	5	(35.7)	42	(36.5)	
RIF	No (%)	13	(92.9)	107	(93.0)	1.000
	Yes (%)	1	(7.1)	8	(7.0)	
Treatment related						
Total dose gonadotropins [IU]	*n*	14		109		
	Mean ± SD	1463.2	± 487.5	1727.0	± 670.2	0.128
Oestrogen level [pg/ml]^c^	*n*	14		115		
	Mean ± SD	1396.5	± 891.8	1643.0	± 844.1	0.161
Number of oocytes	*n*	14		115		
	Mean ± SD	9.1	± 3.6	9.8	± 4.5	0.787
	Total number (min–max)	128	(4–16)	1122	(3–24)	
Fertilisation rate IVF/ICSI [%]	*n*	14		115		
	Mean ± SD	61.7	± 19.6	60.9	± 21.2	0.870
Number of embryos cultured	*n*	14		115		
	Mean ± SD	3.4	± 0.8	3.5	± 0.9	0.666
	Total number (min–max)	48	(2–5)	400	(1–5)	
Treatment method	IVF (%)	12	(85.7)	98	(85.2)	0.783
	ICSI (%)	2	(14.3)	14	(12.2)	
	Splitting (%)	0	(0.0)	3	(2.6)	
Cryopreserved sperm	No (%)	14	(100.0)	114	(99.1)	1.000
	Yes (%)	0	(0.0)	1	(0.9)	
Stimulation protocol	Antagonist (%)	13	(92.9)	89	(77.4)	0.298
	Agonist (%)	1	(7.1)	26	(22.6)	
Calcium ionophore	No (%)	14	(100.0)	113	(98.3)	1.000
	Yes (%)	0	(0.0)	2	(1.7)	
Pregnancy test	Negative (%)	6	(54.5)	47	(48.0)	0.757
	Positive (%)	5	(45.5)	51	(52.0)	

*AMH* anti-Müllerian hormone, *BMI* body mass index, *Max* maximum, *Min* minimum, *RIF* recurrent implantation failure, according to ESHRE (Cimadomo et al. [18]), *SD* standard deviation

^a^The *P* values presented were calculated using the Mann–Whitney *U* test for continuous variables and Fisher’s exact test for categorical variables. Bold values indicate a statistically noticeable difference between groups

^b^Number of previously transferred embryos that did not implant

^c^Oestrogen level prior to oocyte retrieval

There was no evidence of difference in all collected treatment related characteristics between the PCOS and non-PCOS groups (Table 1). Although the pregnancy rate was lower in the PCOS group compared to the control group, this difference was not statistically noticeable (45.5% vs 52.0%, *P* = 0.757, Table 1).

### Morphokinetics

Mean and median were calculated for each morphokinetic value (Table 2). The results of the GLMM did not show any statistically noticeable influence of PCOS on absolute morphokinetic time points (Table 3). However, there was evidence that PCOS influences several relative morphokinetics. The relative time intervals s2 (*P* = 0.013), s3 (*P* = 0.032) and ECC3 (*P* = 0.043) were noticeably longer in embryos from the PCOS group [15]. The relative time ratio CS2-8 was noticeably lower (*P* = 0.003) and CS2-4 higher (*P* = 0.001) in embryos from the PCOS group (Table 3) [15].

**Table 2:** Comparison of morphokinetics in PCOS and control group

Morphokinetics^a^ *n* (PCOS/control)		PCOS group		Control group		*P* value^b^ (*t* test)	*P* value^b^ (*U* test)
t2-tPNf [h]	M (± SD)	3.1	(± 1.8)	3.2	(± 2.0)	0.838	0.422
n (46/354)	Md (min–max)	2.8	(1.8–13.9)	2.7	(0.2–18.3)		
t3-tPNf [h]	M (± SD)	13.4	(± 5.6)	14.5	(± 5.8)	0.225	0.641
n (41/341)	Md (min–max)	14.0	(3.0–31.4)	13.8	(2.3–60.4)		
t4-tPNf [h]	M (± SD)	17.8	(± 8.3)	16.6	(± 6.5)	0.267	0.279
n (40/325)	Md (min–max)	14.9	(9.3–57.1)	14.7	(3.0–51.5)		
t5-tPNf [h]	M (± SD)	28.2	(± 11.3)	27.7	(± 8.2)	0.754	0.807
n (33/311)	Md (min–max)	26.7	(13.3–59.9)	26.9	(12.0–78.4)		
t6-tPNf [h]	M (± SD)	30.1	(± 9.3)	30.0	(± 8.8)	0.987	0.586
n (31/297)	Md (min–max)	28.5	(14.3–50.3)	28.5	(12.8–75.2)		
t7-tPNf [h]	M (± SD)	34.9	(± 10.8)	32.4	(± 8.4)	0.123	0.155
n (31/284)	Md (min–max)	32.5	(22.3–64.3)	29.9	(13.0–74.4)		
t8-tPNf [h]	M (± SD)	39.5	(± 11.8)	35.4	(± 9.6)	**0.035**	0.083
n (29/269)	Md (min–max)	37.0	(26.8–66.9)	32.5	(14.8–78.0)		
t9 + -tPNf [h]	M (± SD)	45.3	(± 10.2)	45.5	(± 8.5)	0.917	0.945
n (21/223)	Md (min–max)	44.7	(29.5–65.8)	44.9	(25.2–79.0)		
tSC-tPNf [h]	M (± SD)	57.1	(± 8.8)	52.2	(± 9.9)	**0.029**	**0.030**
n (21/234)	Md (min–max)	58.6	(41.3–75.9)	51.6	(26.1–79.0)		
tM-tPNf [h]	M (± SD)	63.6	(± 8.2)	61.1	(± 9.8)	0.261	0.271
n (20/220)	Md (min–max)	64.0	(53.0–83.6)	60.3	(33.2–93.0)		
tSB-tPNf [h]	M (± SD)	72.7	(± 7.4)	70.2	(± 7.8)	0.182	0.222
n (18/178)	Md (min–max)	70.7	(62.3–88.4)	69.9	(43.7–92.6)		
tEB-tPNf [h]	M (± SD)	81.0	(± 6.6)	78.1	(± 6.7)	0.170	0.172
n (11/128)	Md (min–max)	81.2	(70.4–93.5)	77.2	(63.4–93.6)		
s2 [h]	M (± SD)	4.4	(± 5.7)	2.4	(± 4.3)	**0.034**	** < 0.001**
n (41/344)	Md (min–max)	1.3	(0.2–25.7)	0.7	(0.0–26.5)		
s3 [h]	M (± SD)	13.1	(± 10.7)	9.3	(± 8.4)	**0.024**	0.073
n (30/288)	Md (min–max)	12.8	(0.7–41.3)	5.8	(0.3–48.0)		
cc2a [h]	M (± SD)	10.2	(± 5.8)	11.4	(± 5.3)	0.166	0.595
n (42/359)	Md (min–max)	11.1	(0.0–28.4)	11.0	(0.0–57.5)		
cc2b/ECC2 [h]	M (± SD)	14.6	(± 8.0)	13.4	(± 5.6)	0.254	0.245
n (41/343)	Md (min–max)	12.4	(5.3–54.1)	11.8	(0.2–46.8)		
cc3a [h]	M (± SD)	10.5	(± 7.2)	11.6	(± 5.8)	0.400	0.625
n (34/330)	Md (min–max)	11.4	(0.0–26.4)	12.1	(0.0–52.9)		
cc3b [h]	M (± SD)	14.0	(± 6.3)	14.3	(± 6.3)	0.824	0.704
n (32/316)	Md (min–max)	13.9	(1.0–26.2)	13.6	(0.0–49.5)		
cc3c [h]	M (± SD)	18.8	(± 7.4)	17.0	(± 5.9)	0.111	0.189
n (32/303)	Md (min–max)	16.3	(8.5–40.5)	15.3	(3.5–43.1)		
cc3d/ECC3 [h]	M (± SD)	23.7	(± 10.5)	20.3	(± 7.8)	0.095	0.149
n (30/288)	Md (min–max)	20.8	(12.8–48.7)	17.7	(9.9- 58.5)		
CS2-8	M (± SD)	0.6	(± 0.3)	0.7	(± 0.2)	**0.020**	**0.015**
n (30/287)	Md (min–max)	0.6	(0.0–1.0)	0.8	(0.0–1.0)		
CS4-8	M (± SD)	0.5	(± 0.3)	0.4	(± 0.3)	**0.046**	0.098
n (30/288)	Md (min–max)	0.5	(0.0–1.0)	0.3	(0.0–1.0)		
CS2-4	M (± SD)	0.3	(± 0.3)	0.1	(± 0.2)	**0.013**	** < 0.001**
n (41/343)	Md (min–max)	0.1	(0.0–1.0)	0.1	(0.0–1.0)		
DR	M (± SD)	1.0	(± 1.7)	1.1	(± 1.9)	0.714	**0.002**
n (34/328)	Md (min–max)	0.7	(0.0–10.0)	0.8	(0.0–22.0)		

*h* hours, *M* mean, *Max* maximum, *Md* median, *Min* minimum, *SD* standard deviation

^a^Morphokinetics are defined according to Ciray et al. [4] and Cetinkaya et al. [8]

^b^*P* values from *t* test and *U* test were calculated for all parameters to ensure robustness. *P* values < 0.05 are shown in bold and indicate statistically noticeable differences in an explorative context

**Table 3:** Results of the Generalised Linear Mixed Model (GLMM) assessing the influence of PCOS on morphokinetics

Morphokinetics^a^	*n* (of 448)	Coefficient	SE	*P* value^b^
t2-tPNf	400	−0.059	0.380	0.877
t3-tPNf	382	−1.190	1.053	0.259
t4-tPNf	365	1.056	1.255	0.401
t5-tPNf	344	0.246	1.788	0.891
t6-tPNf	328	0.036	1.780	0.984
t7-tPNf	315	2.446	1.781	0.171
t8-tPNf	298	4,023	2.180	0.066
t9 + -tPNf	244	−0.329	2.327	0.888
tSC-tPNf	255	4.381	2.735	0.110
tM-tPNf	240	1.653	2.794	0.555
tSB-tPNf	196	2.266	2.317	0.329
tEB-tPNf	139	2.684	2.345	0.254
s2	385	1.953	0.780	**0.013**
s3	318	3.864	1.793	**0.032**
cc2a	401	−1.224	0.953	0.200
cc2b (ECC2)	384	1.003	1.064	0.347
cc3a	364	−1.088	1.106	0.326
cc3b	348	−0.260	1.172	0.824
cc3c	335	1.784	1.213	0.142
cc3d (ECC3)	318	3.516	1.727	**0.043**
CS2-8	317	−0.143	0.049	**0.003**
CS4-8	318	0.108	0.055	0.052
CS2-4	384	0.141	0.043	**0.001**
DR	362	−0.123	0.340	0.718

*SE* standard error

^a^Morphokinetics are defined according to Ciray et al. [4] and Cetinkaya et al. [8]

^b^*P* values were calculated using a Generalised Linear Mixed Model (GLMM). *P* values < 0.05 are shown in bold and are considered statistically noticeable in an explorative context

The results of the *t* test and Mann–Whitney *U* test confirmed the evidence of differences in s2, s3, CS2-8 and CS2-4 between PCOS and non-PCOS groups (Table 2). In the *t* test and/or Mann–Whitney *U* test, differences between the groups were also found for t8-tPNf, tSC-tPNf, CS4-8 and DR (Table 2).

## Discussion

This study demonstrated an influence of PCOS on relative embryo morphokinetics, in terms of poorer cleavage synchronicity. To the best of our knowledge, this is the first study to include relative ratios in a morphokinetic study on PCOS. The study’s strengths include the exclusion of confounding comorbidities, constant laboratory conditions and retrospective re-annotation by a single observer. The latter reduced the inter- and intra-observer variability, which is typical of TLI annotations [19]. A consecutive inclusion of all eligible patients at our clinic during the study period minimised the selection bias. Moreover, the PCOS and non-PCOS groups showed relatively equal clinical characteristics. The differences in female patients’ age and serum AMH levels are regarded as characteristic of the condition. High serum AMH levels are typical of PCOS [20, 21]. The early onset of PCOS might lead to infertility treatment at younger ages compared to other causes (e.g. poor ovarian reserve). Since advanced maternal age was found to delay later blastocyst timings, but did not affect early cleavage synchronicity, the age difference was not expected to influence relative timings [22–24]. Instead, our results suggest an age-independent negative PCOS influence.

This study’s limitations include the retrospective design and the small PCOS cohort, potentially resulting in an unbalanced design compared to the control group. However, the main statistical strength of this study was the utilisation of a GLMM, with patient ID as a random effect. This hierarchical approach accounted for the clustering of embryos within patients and ensured that results reflected group-wide trends rather than being driven by individual outliers [15]. Limited by the lack of prior data for power and sample size calculation, our findings are explorative. Nonetheless, to ensure robustness, we performed sensitivity analyses using the *t* test and the Mann–Whitney *U* test, which confirmed the trends observed for CS2-8​, CS2-4​ and s2​. The latter, being a non-parametric test, also accounted for small group sizes. Nevertheless, larger multi-centre trials are required to confirm these findings in more balanced populations.

The four ratios published by Cetinkaya et al. represent cleavage synchronicity in embryonic cell cycles. Ideally, identical cell cycle regulation in sister blastomeres would result in complete synchronicity of cleavage and very short 3-, 5-, 6- and 7-cell stages [8]. Consequently, CS2-8 ideally tends towards 1, whereas CS4-8 and CS2-4 ideally tend towards 0 [8]. In this study, the ratios in PCOS embryos showed the expected tendencies towards impaired cleavage synchronicity, possibly implicating poorer developmental potential. PCOS led to a lower CS2-8 (*P* = 0.003) and higher CS2-4 (*P* = 0.001). The latter is linked to the prolongation of s2 (*P* = 0.013), which constitutes the numerator of CS2-4 [15]. As Meseguer et al. showed that embryos with a short interval s2 had significantly higher implantation rates [6], these altered morphokinetics in PCOS could suggest lower implantation potential. Regarding CS4-8, there was a trend in PCOS embryos towards a higher ratio in this study (*P* = 0.052). In contrast to our findings, the studies by Sundvall et al., Tam Le et al., and Wissing et al. found no significant difference in s2 between PCOS and controls [11, 12, 14]. In addition, s3 (*P* = 0.032) was found to be longer in this study, unlike in the report by Sundvall et al., where it remained unaffected [12, 15].

Whilst the primary GLMM analysis in the present study revealed no influence of PCOS on absolute times, the additional tests indicated two delayed timings: t8-tPNf in the *t* test (*P* = 0.035) and tSC-tPNf in both *t* and Mann–Whitney *U* tests (*P* = 0.029, *P* = 0.030). Wissing et al. reported significantly later absolute times for pronuclear breakdown, t2, t3, t4 and t7 in hyperandrogenic PCOS compared to non-PCOS embryos [11]. Delayed morphokinetic time points are possible negative indicators of developmental potential and implantation [7, 25]. In contrast to the findings by Wissing et al., *earlier* absolute timings in PCOS embryos were observed by Sundvall et al. (tSC, tM) and Chappell et al. (t7, t8 and t9) [12, 13]. Tam Le et al. did not find any different timings up to the 6-cell stage between PCOS and non-PCOS [14]. Whilst the small sample size of the PCOS group in the present study precluded a sub-analysis, some studies differentiated between androgen levels. Wissing et al. identified a delay in early timings only in embryos from patients with hyperandrogenic PCOS, whereas there were no significant differences between embryos from normoandrogenic PCOS patients and non-PCOS controls. They suggested hyperandrogenaemia in PCOS as a possible driver of early morphokinetic alterations from pronuclear breakdown on, potentially caused by the metabolic influence of high androgen levels on oocyte maturation [11, 26]. On the other hand, a sub-analysis of hyperandrogenic PCOS in the study by Chappell et al. could not reproduce these findings, observing earlier timings for later cleavages (t5, t6, t7, t8, t9 and tM) [13].

These inconsistent findings on absolute morphokinetics are striking and might partially result from diverse study designs and cohorts. This divergence underscores the need for future research to focus on relative morphokinetics, and especially ratios, rather than absolute time points alone.

PCOS involves potential endocrine, metabolic and inflammatory changes that may influence the oocyte and embryo development [27]. Elevated inflammatory mediators like Tumour Necrosis Factor alpha in PCOS patients could decrease oocyte quality [28–31]. Moreover, oocyte and embryo development could not only be influenced by androgen excess, but also by elevated AMH levels and oxidative stress in obese PCOS patients [32–35]. Furthermore, altered gene expression in oocytes of PCOS patients might affect the developmental potential of oocytes and embryos [36–38]. The embryo depends on maternal messenger ribonucleic acid (mRNA) transcripts until activation of the embryonic genome takes place at around the 8-cell stage [11, 36]. Since relative morphokinetic ratios represent early cleavage synchronicity up to this stage, their alteration in PCOS may reflect a dependency on maternal factors.

Whilst Meseguer et al. showed that relative morphokinetics in prediction models for implantation rates might be superior to absolute time points [6], the effect of their alterations on clinical outcomes of IVF/ICSI treatments remains to be fully elucidated. Identifying morphokinetic parameters predictive for implantation, pregnancy, or live birth rates could help integrating multiple morphokinetic with morphological criteria in embryo classification models for choosing the most viable embryo. At this point, the growing importance of artificial intelligence (AI) for automated annotation, for embryo grading and selection should be emphasised [39]. AI-based automated annotation could minimise inter- and intra-observer variability and increase the potential predictive value of embryo morphokinetics [40]. Regarding deep learning models, full insight into internal selection algorithms is not possible, but future research might reveal if correlations between cleavage synchronicity and AI-based decision-making for embryo selection exist.

Regardless of their predictive value for clinical outcomes, relative embryo morphokinetics provide profound insights into embryo development and particularly cleavage synchronicity. Calculating these variables from routinely assessed absolute time points requires no significant additional effort. Although relative intervals such as s2 and s3 are recognised as part of good practice and are integrated into automated annotation systems, they have yet to be consistently adopted in clinical routines [5]. Furthermore, the four ratios by Cetinkaya et al. have not yet gained a role in clinical practice. The present study demonstrates that relative morphokinetics, especially ratios, could be superior to absolute times in identifying PCOS-related alterations. In addition, as relative ratios enable identification of impaired cleavage patterns on day 3, they are of special interest to laboratories performing cleavage stage transfers [8]. Their routine implementation would facilitate further studies on clinical outcomes. How PCOS affects embryo development is also of high interest to patients themselves and could influence patient counselling.

## Conclusion

In this study, statistically noticeable changes in relative morphokinetic time intervals and ratios, associated with impaired cleavage synchronicity, were found in PCOS embryos compared to those from patients with uterine factor, tubal factor or idiopathic infertility. This study is of special relevance, as, to the best of our knowledge, it is the first to examine the influence of PCOS on relative morphokinetic ratios. These findings are important because morphokinetic changes in PCOS embryos were exclusively detected in relative morphokinetics, whereas absolute time points remained unchanged. Incorporating these potentially superior relative morphokinetics, especially time ratios, into clinical practice may provide more detailed information on impaired embryo development. Whether relative morphokinetic alterations in PCOS embryos, in terms of reduced cleavage synchronicity, indicate poorer developmental potential and clinical outcomes needs to be investigated further in future studies.

## Data Availability

The datasets used and/or analysed during the current study are available from the corresponding author on reasonable request.
